# Malingering in ADHD behavioral rating scales: recommendations for research contexts

**DOI:** 10.3389/fpsyt.2025.1532807

**Published:** 2025-01-24

**Authors:** Marius Grandjean, Shachar Hochman, Raja Mukherjee, Roi Cohen Kadosh

**Affiliations:** ^1^ School of Psychology, University of Surrey, Guildford, United Kingdom; ^2^ Psychological Sciences Research Institute, UCLouvain, Louvain-la-Neuve, Belgium; ^3^ School of Medicine, University of Surrey, Guildford, United Kingdom

**Keywords:** ADHD (attention deficit hyperactivity disorder), malingering and feigning detection, research, diagnostic, behavioral rating scale

## Introduction

Attention Deficit Hyperactivity Disorder (ADHD) is a neurodevelopmental disorder characterized by persistent patterns of inattention, distractibility, hyperactivity, and impulsivity, which interfere with functioning or development (1). ADHD is associated with an elevated risk of other mental health disorders and adverse outcomes such as educational underachievement, employment difficulties, interpersonal relationship challenges, and potential involvement in criminal activities (2). These far-reaching impacts make accurate and reliable ADHD assessments critical for both clinical and research purposes.

Diagnosing ADHD involves various methods, including clinical interviews, continuous performance tests, and behavioral rating scales. Best practices recommends triangulating information via a comprehensive diagnostic approach that synthesizes information from multiple sources, such as structured interviews, cognitive assessments, and behavioral rating scales (3, 4). However, in research contexts—particularly studies exploring new treatment approaches—behavioral rating scales are often the preferred outcome measure due to their cost-effectiveness, ease of administration, and accessibility (5–10).

While this pragmatic choice is often driven by resource constraints that limit the feasibility of more comprehensive assessment procedures (11), it highlights a critical responsibility for researchers: ensuring the data collected through these scales accurately represent the genuine experiences of respondents. Selecting the appropriate scale is not just a matter of practicality—it is foundational to producing reliable, meaningful research outcomes. However, behavioral rating scales are not without their challenges. Their inherent subjectivity makes them vulnerable to feigned or exaggerated symptom reporting, which can compromise the validity of findings, and hinder scientific progress.

To mitigate these risks, researchers need to remain vigilant about advancements in ADHD assessment methodologies, particularly the development of tools designed to detect invalid or exaggerated symptom presentations. These tools play a crucial role in distinguishing genuine cases from noncredible reports, ensuring that research findings are both reliable and meaningful. Without this level of scrutiny, studies risk being undermined by data that fail to accurately represent the true experiences of participants.

In this opinion paper, we aim to provide researchers with an overview of the most widely used ADHD rating scales, focusing specifically on their capacity to detect malingering—‘the intentional production of false or grossly exaggerated physical or psychological symptoms, motivated by external incentives’ (1). Additionally, we offer practical recommendations to guide researchers in selecting assessment tools that maximize diagnostic accuracy, enhancing the reliability and validity of their research. By addressing the challenges of malingering and invalid symptom reporting, we aim to contribute to the development of more robust ADHD evaluation strategies to allow the needed scientific progress.

## Why should we care about malingering?

Diagnosing ADHD presents unique challenges, primarily due to the commonality of its symptoms—such as inattention, impulsivity, and hyperactivity—among the general population (12). These symptoms are often encountered to varying extents, complicating the differentiation between genuine cases and instances of feigned or malingered presentations. Moreover, the ease with which ADHD symptoms can be feigned introduces another layer of complexity to the diagnostic process (13).

In recent years, the complexity of ADHD presentations and the prevalence of potential comorbidities have further extended the diagnostic challenges beyond intentional malingering to include unintentional misdiagnosis. Both malingering and misdiagnosis highlight the critical need for accurate assessment measures in ADHD diagnosis. The vulnerability of behavioral rating scales to falsification is a particularly concerning issue, given their subjective nature (14–17).

The susceptibility of questionnaires to feigned responses is heightened by the diverse motivations individuals may have for fabricating ADHD symptoms. These include attaining social acceptance, gaining access to ADHD medications, or even enhancing academic performance (14, 18, 19). The non-specificity of the ADHD symptoms outlined in the DSM-5 further facilitates the feigning of symptoms, especially in adults, particularly among college students who may attempt to manipulate their presentation during assessments (13, 15). Alarmingly, the prevalence of feigned ADHD symptoms among students ranges from 5% to 50% (20). However, this critical issue remains largely overlooked in the literature. Specifically, only half of the recent reviews on rating scales address this problem, and even within these discussions, the topic is often treated superficially, with only brief or incidental mention (3, 21–23).

Recognizing the potential consequences of ignoring feigned ADHD symptoms in research contexts is paramount. Neglecting this issue could lead to the mismanagement of resources, erroneous conclusions, and the failure of trials, development of biomarkers, or replications, as findings may be based upon inaccurate diagnoses. Therefore, it is essential for researchers to detect feigned ADHD symptoms when utilizing behavioral rating scales to enhancing the integrity and reliability of their findings.

## Addressing feigned ADHD symptoms in assessments

Among the most commonly used behavioral rating scales in adult ADHD assessment are the Adult ADHD Self-Report Scale (ASRS; 11), the Brown Attention-Deficit Disorder Scale (BADDS; 24), the Wender Utah Rating Scale (WURS; 25), the Barkley Adult ADHD Rating Scale (BAARS-IV; 26) and the Conners’ Adult ADHD Rating Scales (CAARS; 27). Publicly available scales like the ASRS assess ADHD symptoms outlined in the DSM-4 (28) while the WURS retrospectively evaluates childhood ADHD symptoms. Commercial tools such as the BAARS and the CAARS offer more comprehensive assessments. Notably, the CAARS and BAARS-IV provide self- and observer-reported versions, enhancing reliability. These scales are valued for their ease of administration and ability to measure ADHD symptom severity across various domains of functioning (see **Table 1**). However, their reliance on self-reported data, without accounting for feigned symptoms, increases the likelihood that the score in these questionnaires could include intentional exaggeration or falsification of symptoms.

**Table 1:** Description of the most widely used behavioral rating scales for diagnosing adult ADHD.

Scale	Description	Malingering detection capacity	Strengths	Limitations
ASRS	Self-report scale with two versions: a 6-item screener and an 18-item full version, both assessing ADHD symptoms based on DSM-IV criteria.	No	Brief and easy to administer. Widely used and validated.	Does not comprehensively assess functional impairment.
BADDS	Self-report scale consisting of 40 items, assessing DSM-IV ADHD symptoms and additional executive function impairments often associated with ADHD but not included in DSM diagnostic criteria.	No	Assesses a broader range of ADHD-related difficulties, including executive function deficits.	Not as widely used or validated as some other scales.
WURS	Retrospective self-report scale for childhood ADHD symptoms. Two versions are available: the 61-item version (WURS-61), providing comprehensive coverage of symptoms and potential confounders, and a shorter 25-item version (WURS-25) for greater efficiency.	No	Useful for gathering information about childhood history of ADHD symptoms. The long version offers a very comprehensive assessment.	Relies on retrospective recall, which can be unreliable. Not designed for assessing current symptoms.
BAARS-IV	Self- or other-report scale based on DSM-IV criteria. Includes two forms: one for current symptoms (30 items) and one for childhood symptoms (20 items). Both forms have quick screen versions, with 8 items for current symptoms and 6 items for childhood symptoms.	No	Allows for both self and other reports, providing a more comprehensive view. Includes both current and retrospective symptom assessment.	
CAARS	Self- or other-report scale based on DSM-IV criteria (CAARS-S or CAARS-O). Available in three versions: a 26-item short form (CAARS-S:S), a 30-item screening form (CAARS-S:SV), and a 66-item comprehensive form (CAARS-S:L).	Yes	Allows for both self and other reports. Comprehensive versions provide detailed information.	The comprehensive form is more time-consuming to administer than shorter scales.

ASRS, ADHD Self-Report Scale; BADDS, Brown Attention-Deficit Disorder Scale; WURS, Wender Utah Rating Scale; BAARS-IV, Barkley Adult ADHD Rating Scale – Fourth Edition; CAARS, Conner’s Adult ADHD Rating Scales.

Fortunately, recent years have seen a surge in the development of tools aimed at identifying invalid ADHD symptom reports (29–31). These tools, commonly referred to as symptom validity tests (SVTs), can be incorporated into existing scales or used as standalone measures. In contrast with the scales previously mentioned, the CAARS stands out as the sole scale currently featuring two embedded validity indexes^1^, the CAARS Infrequency Index (CII; 32) and the Exaggeration Index (EI; 33). The CII consists of items that are rarely endorsed by individuals with ADHD or by healthy controls, making it highly effective at identifying noncredible symptom reporting when responses exceed a specific threshold. The EI, on the other hand, combines items from the CAARS with additional items adapted from the Dissociative Experiences Scale (DES; 34), all of which are infrequently endorsed by individuals with genuine ADHD. A third validity index, the ADHD credibility index (ACI; 35) is still under development. The ACI uses ADHD-specific items designed to capture various patterns of noncredible symptom reporting. Together, these indexes help determine whether an individual’s symptom reports align with expected behavioral patterns, providing a robust method for detecting malingering.

In addition to these embedded SVTs, there is also an increasing demand for standalone SVTs specifically designed to distinguish between genuine and feigned ADHD symptoms. Notable examples include the ADHD Symptom Infrequency Scale (ASIS; 36), which consists of two subscales: the ADHD subscale (aligned with DSM-5 diagnostic criteria) and the Infrequency subscale (designed to identify symptoms more likely to be endorsed by individuals feigning ADHD). Another scale is the Multidimensional ADHD Rating Scale (MARS; 37), which includes three categories of items: symptom items, impairment items, and symptom-validity items. The MARS also incorporates “catch” items to assess the test-taker’s effort and attention during the assessment. Although these scales show promise, further validation is needed to confirm their reliability and accuracy.

One significant advantage of using embedded SVTs over standalone measures is that they make the detection strategy less transparent to individuals who might attempt to feign symptoms. The subtlety of embedded SVTs minimizes the chances of test-takers altering their responses when they are aware of the detection process (32).

## Discussion

The critical issue of feigning in ADHD assessments has long been overlooked, despite its significant impact on both clinical practice and research. Accurate assessments are essential for diagnosis and treatment planning, and it is crucial that the detection of feigned responses becomes a standard part of all behavioral rating scale protocols. As the field moves toward developing novel methods for identifying feigned responses, we expect substantial improvements in accuracy and precision. However, until these advancements become widely available, it is vital to continue using well-established tools that have proven their effectiveness over time. While the scope of providing recommendations on using SVTs in clinical settings is outside the scope of the current paper, we refer the reader to Marshall et al. (3), whose review also underscores their relevance to diagnostic procedures.

**Figure 1:**
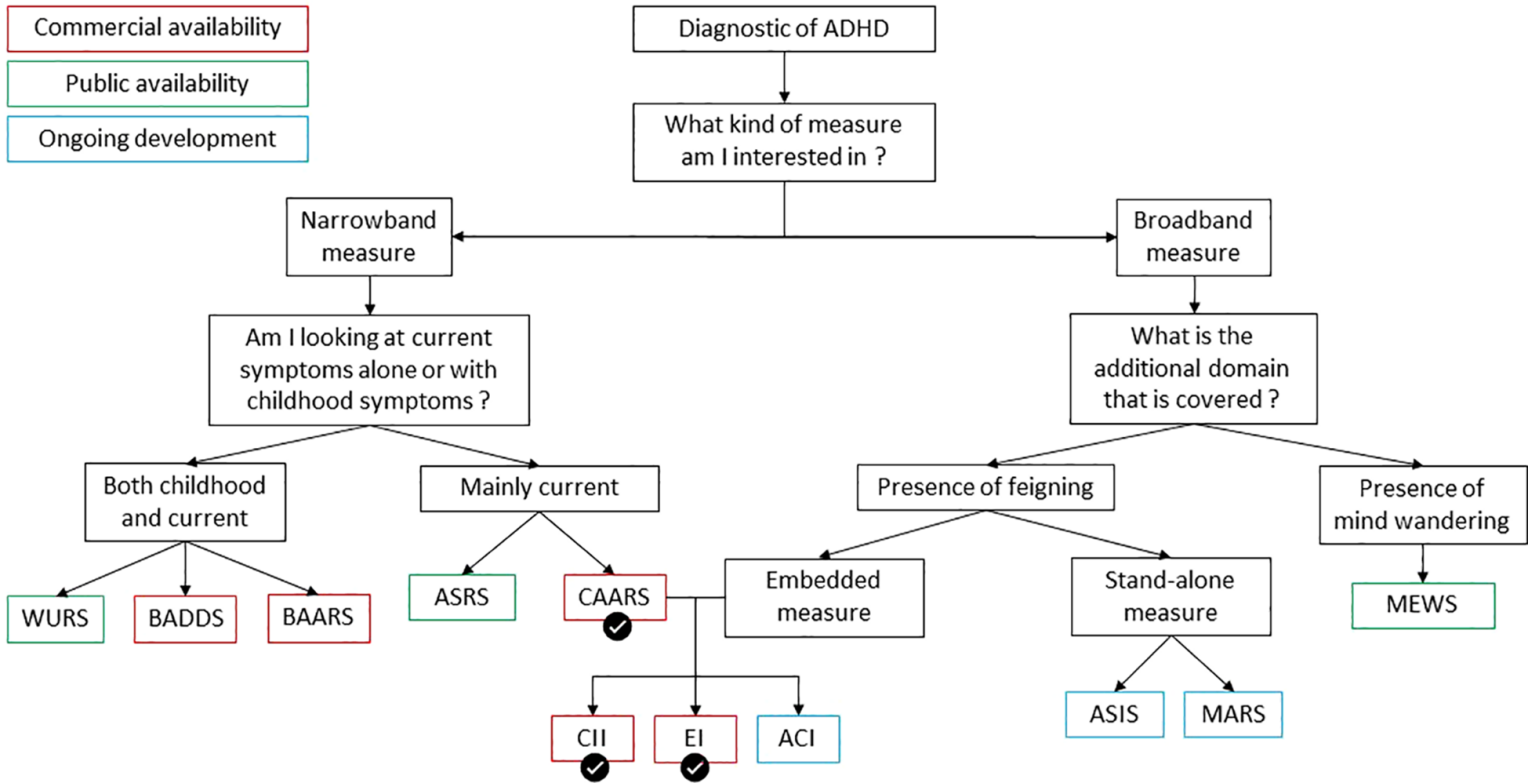
Flowchart of ADHD assessment tools based on availability, symptoms measured and detection of feigning. Measures are color-coded for their availability status—green for publicly available, red for commercially available, and blue for those under development. Recommended measures are indicated with a checkmark. The CII and EI are marked in red, as their use requires the CAARS-S. Narrowband measures target specific ADHD symptoms, while broadband measures assess a wider range of behaviors. Key abbreviations include: WURS (Wender Utah Rating Scale), BADDS (Brown Attention-Deficit Disorder Scale), ASRS (ADHD Self-Report Scale), CAARS (Conner’s Adult ADHD Rating Scales), CII (CAARS Infrequency Index), ACI (ADHD Credibility Index), ASIS (ADHD Symptom Infrequency Scale), BAARS-IV (Barkley Adult ADHD Rating Scale – Fourth Edition), EI (Exaggeration Index), MEWS (Mind Excessively Wandering Scale), and MARS (Multidimensional ADHD Rating Scale).

Based on current evidence, we strongly recommend using the long version of the CAARS self-report form (CAARS-S:L), due to its proven accuracy and widespread acceptance in the field (3, 23). The CAARS has consistently been identified as one of the most reliable tools for assessing ADHD symptoms. One of its key strengths is its ability to detect feigned responses, with its embedded validity indexes—the CII and EI—which help to identify invalid symptom reporting. We recommend using both of them as employing multiple SVTs effectively lowers the incidence of false positives in malingering evaluations (17). Additionally, unlike other scales such as the WURS or ASRS, the CAARS covers a broader range of ADHD symptoms, including those not specifically outlined in the DSM-5, enhancing its diagnostic utility. Moreover, the CAARS is particularly advantageous in monitoring treatment efficacy, as it tracks both the presence and severity of ADHD symptoms over time, unlike the WURS, which only addresses historical symptoms. Future research should clarify how these tools, including the ADHD Credibility Index (ACI), be further verified and customized for usage in varied demographics and circumstances. To conclude, we have outlined key assessment tools to address the challenge of feigned or malingering ADHD symptoms, considering their scope, target populations, usage terms, and effectiveness. **Figure 1** summarizes our recommendations. Although the CAARS is a commercial tool and not freely available, its comprehensive coverage of ADHD symptoms, ability to detect feigned responses through embedded validity indexes, and its utility in monitoring both symptom severity and treatment efficacy make it an invaluable resource for accurate and reliable ADHD assessment in research settings.

## Footnotes

1 These two indexes were developed to be used with the CAARS-S:L. The CAARS-S:S and CAARS-S:L also have an Inconsistency Index but it only measures the consistency at which an individual is reporting similar symptoms.
